# Changes in the Structural Composition and Moisture-Adsorption Properties of Mechanically Rolled Bamboo Fibers

**DOI:** 10.3390/ma15103463

**Published:** 2022-05-11

**Authors:** Wenjuan Zhao, Jian Zhang, Wenfu Zhang, Jin Wang, Ge Wang

**Affiliations:** 1 Zhejiang Academy of Forestry, Hangzhou 310023, China; zwj18066088392@163.com (W.Z.); zhjianzj@126.com (J.Z.); whuwj@sina.com (J.W.); 2 College of Furnishings and Industrial Design, Nanjing Forestry University, Nanjing 210037, China; 3 International Centre for Bamboo and Rattan, Beijing 100102, China; wangge@icbr.ac.cn

**Keywords:** bamboo fiber, mechanical rolling, structural composition, chemical compositions, pyrolysis performance, moisture-adsorption properties

## Abstract

The chemical content, mechanical capability, and dimensional stability of bamboo fibers (BFs) are all directly related to the hygroscopic behavior, which is crucial for industrial applications. To support the utilization of BFs, the structural and chemical composition of BFs with different opening times after mechanical rolling were investigated in this study, and the Guggenheim–Anderson–de Boer (GAB) model was selected to predict their moisture-adsorption properties. The results showed that the length and diameter of the fibers gradually decreased with the increase in the number of openings, and the fibers gradually separated from bundles into single fibers. It was also observed that the treated BFs exhibited different equilibrium moisture contents (EMCs). BFs with a smaller number of openings had a higher hemicellulose content and more exposed parenchyma cells on the fibers, which increased the number of water adsorption sites. As the number of openings increased, the parenchyma cells on the fibers decreased, and the lignin content increased, which reduced the number of fiber moisture-adsorption sites and decreased the EMC of the fibers.

## 1. Introduction

Bamboo is composed of a two-phase structure, namely, vascular bundles and parenchyma cells [1]. Bamboo mechanics are mostly governed by bamboo fibers (BFs), which are abundant in vascular bundles. Using physical, chemical, or biological processes to treat the bamboo to remove lignin, pectin, polysaccharides, and other components, the BF is separated from the matrix to yield natural BF. These methods can retain the original microstructure of the bamboo and have the advantages of high specific strength and specific modulus. As such, it can be used in textile materials and fiber-reinforced composites [2,3,4,5].

Presently, the main processing methods of BFs can be broadly classified into physical–mechanical methods and combined physical–biochemical methods. The former obtains coarse BFs by separation, while the latter uses the method of separating fibers from the gum to yield refined BFs [6,7]. Although the fibers obtained by the combined physical–biochemical method have better fineness and softness, this method has a complex treatment process and a high cost. In contrast, natural BFs prepared by physical–mechanical methods are environmentally friendly, efficient, and can be produced industrially, which has become a hot research topic in recent years [8]. The mechanical combing method is the most commonly used method at present, which combs the rolled or milled bamboo material into bundled fibers of uniform size. With the increase in fiber combing time, the monofilament fiber size gradually becomes smaller, and the size variability gradually decreases [9,10]. The BF extracted near the outer layer of bamboo is stiff, large, and yellow, while the BF extracted near the inner layer of bamboo is soft, small, and white [11]. This method can effectively control the fiber size variability, but the low production efficiency destroys the fracture strength of the fibers, which is a difficult problem to solve during the production process. By contrast, the advantage of mechanical rolling and fiber opening is the high production efficiency. In this method, the bamboo is first divided into portions, then laminated several times to weaken the bonding properties between the fibers, which promotes the separation of the fibers from the matrix. Then, the intertwined fiber masses are torn apart and broken loose by a fiber-opening machine [12,13]. Although theoretical studies are still rare, mechanical rolling and fiber-opening technology has a broad application prospect as one of the key technologies for continuous BF preparation [14].

BF, as natural plant material, is highly hygroscopic, and most of its strength and modulus are influenced by the moisture content [15,16]. The main obstacle to the further development of BF in composites is that its hydrophilic nature adversely affects adhesion to the hydrophobic matrix [17]. To reveal the mechanism of moisture absorption, several mathematical models have been developed to describe the hygroscopic isotherms [18,19]. Based on the hygroscopic behavior of bamboo, the Guggenheim–Anderson–de Boer (GAB) model, which is suitable for materials with large variations in the relative humidity range, was selected to analyze the results obtained in this work [20]. Currently, the investigations into the water vapor sorption behavior of BFs are very limited. Therefore, it is necessary to investigate the relationship between BF and moisture-adsorption properties to support and improve the processing and manufacturing of bamboo products.

In this study, BFs of different sizes were processed with the rolling and opening method, and we examined the characterization of BFs after rolling and opening by environmental scanning electron microscopy (SEM), thermogravimetric analysis (TGA), and dynamic vapor sorption (DVS). An understanding of the structural composition and moisture-adsorption properties of BFs can be used to model and optimize the performance of BFs in the subsequent production process.

## 2. Materials and Methods

### 2.1. Materials

Two-to-three-year bamboo species (*Bambusa emeiensis L.C.* *Chia & H.L. Fung*) were harvested in Chengdu, Sichuan Province, China. They are widely distributed in Sichuan Province in China and has the potential for mass acquisition. These bamboos had a diameter at breast height of 60–80 mm and a wall thickness of 4–7 mm.

**Figure 1:**
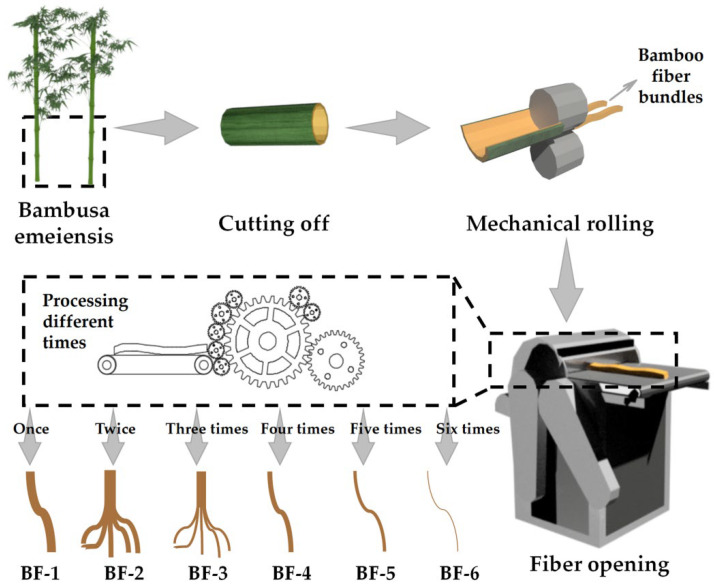
Process for preparing mechanically rolled bamboo fibers.

**Figure 2:**
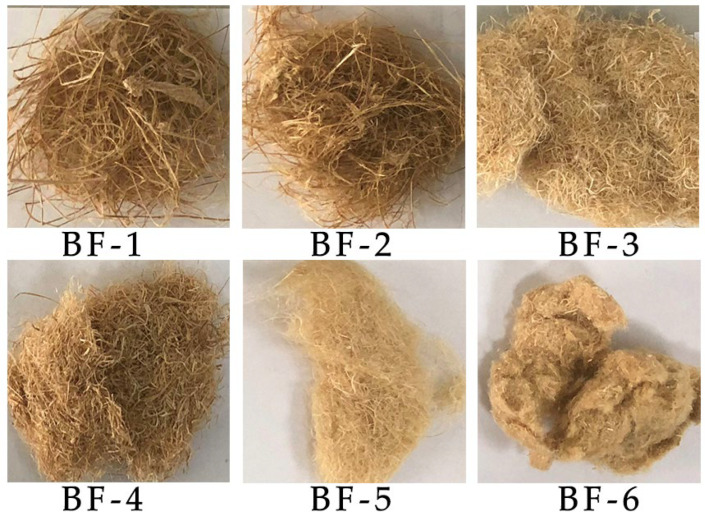
Macroscopic morphology of bamboo fibers with different opening times.

The bamboo material was first cut off and de-jointed into bamboo tubes, then cut into bamboo slices about 10–20 cm long and 2 cm wide. The outer and inner skins of bamboo were reserved. The bamboo slices were processed into soft and loose bamboo fiber bundles by the rolling machine. Bamboo fiber bundles were separated into different forms of fibers after different times of processing by the fiber-opening machine (Figure 1). The whole process took about 18 h. BFs having undergone from one opening step to six opening steps were successively produced according to the number of processing times, with BF-1 representing BFs with one opening process etc., to obtain BFs as follows: BF-1, BF-2, BF-3, BF-4, BF-5, and BF-6 (Figure 2).

### 2.2. Material Chemical Composition

The moisture content, benzyl alcohol extracts, alkali insoluble hemicellulose, and acid insoluble lignin of various materials were measured according to GB 5889-86 [21], and the cellulose content was calculated. Raw bamboo not treated by mechanical rolling and fiber opening was characterized as a control.

### 2.3. Microscopy Observation

The length and diameter of BFs with different opening times were measured using a stereoscopic microscope (Camera Model-Li165; Lumenera, Ottawa, ON, Canada), and thirty random samples from each category were selected. The microstructure of the samples was observed using an environmental scanning electron microscope (FEG-ESEM-XL30; FEI Company, Hillsboro, OR, USA).

### 2.4. Pyrolysis Performance

A thermogravimetric analyzer (TA-500; TA Instruments, New Castle, DE, USA) was used to analyze the pyrolytic properties of the treated and untreated specimens. The specimens were air-dried before the test, and about 10 mg of the specimens was weighed during the test. The specimens were placed in the sample tray, and the heating rate was set to 20 °C/min. The temperature band was from 30 °C to 850 °C, and the data from 30 °C to 800 °C were taken for analysis. The medium atmosphere was high-purity nitrogen, and the airflow rate was 40 mL/min.

### 2.5. Moisture-Adsorption Properties

The moisture-adsorption isotherms of treated and untreated materials were measured using a DVS Resolution dynamic moisture-adsorption instrument (Surface Measurement Systems Ltd., London, UK) in an atmosphere of high-purity nitrogen; then, we analyzed the data curves to determine their moisture-adsorption characteristics. The instrument consisted of a microbalance with two platinum sample crucibles and a humidification system in a temperature-controlled chamber. One of the crucibles was used as a reference, and the other crucible contained the sample to be tested and analyzed. Experimental temperature, 25 ± 0.02 °C. Instrument parameters: balance accuracy, 0.1 μg; target humidity accuracy, ±0.1%. As the DVS technique has been reported to yield highly reproducible data, in this study, only one measurement was reported for each specimen [20,22].

#### 2.5.1. Material Moisture-Adsorption Isotherm Test

Specific test process: Keep the temperature constant during the test; control each sample with a mass of 30–50 mg; place it in the weighing bottle; bake it until it is completely dry. Write the instrument test method (set the step mass change rate and relative humidity gradient). In this study, set the step mass change rate < 0.001%/min and relative humidity 0–95%; the humidity gradient is 0, 10, 20, 30, 40, 50, 60, 70, 80, and 90 and 95%. Conduct three repeated experiments.

Instrument loading test method: add the dried sample; read the mass of the sample; run the test method; automatically collect data every 0.01 min during the test. Record the running time, relative humidity and sample mass throughout the isotherm run. The equilibration time is defined as the time required for the sample to reach EMC at a given relative humidity, and this datum is available after the DVS analysis [23].

#### 2.5.2. Model Analysis of Moisture-Adsorption Characteristics

The GAB equation can be expressed as shown in Equation (1) to determine the coefficient of determination (R2) and the sum of squares of residuals (RSS) of the fitting model [24,25]. When the R2 value is higher and close to 1, and the RSS value is smaller and close to 0, the model is judged to be a better fit.

W_e_ = (W_0_ C K a_w_)/[(1 − K a_w_) (1 − K a_w_ + K C a_w_)] (1)

where W_e_ is the equilibrium moisture content (EMC), W_0_ is the monolayer moisture content, a_w_ is the water activity, and C and K are the model constants that are related to monolayer and multilayer properties.

## 3. Results and Discussion

### 3.1. Structural Analysis

**Figure 3:**
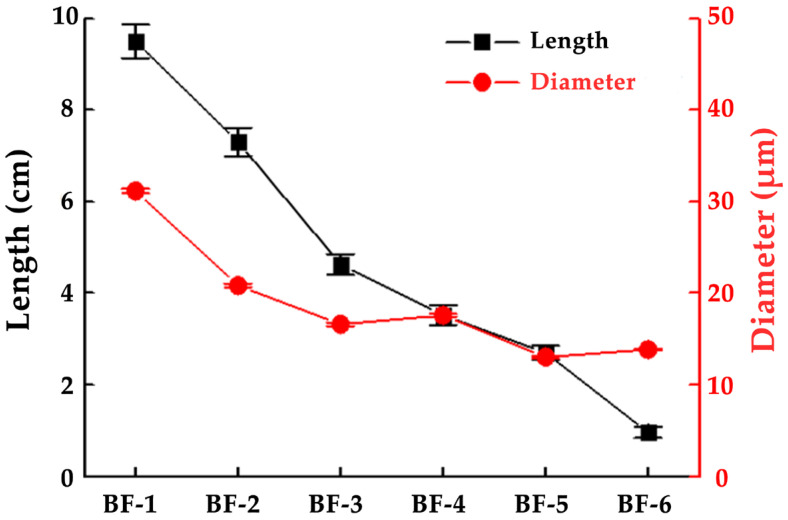
The size of bamboo fibers with different opening times.

**Figure 4:**
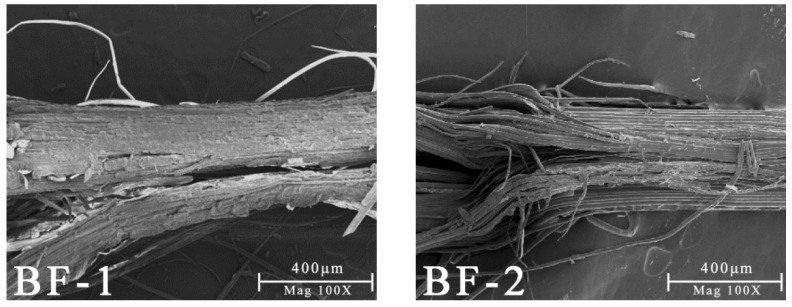

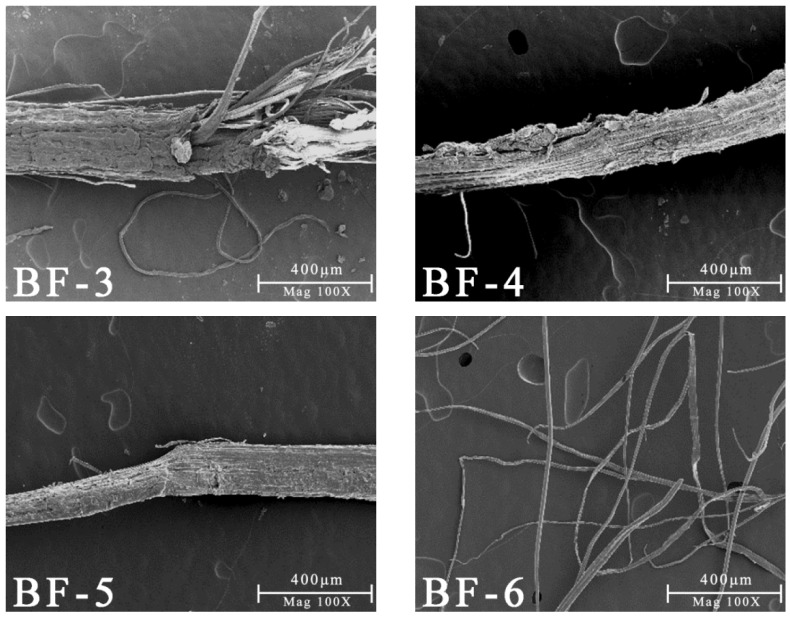
Microscopic morphology of bamboo fibers with different opening times.

As shown in Figure 3, the length and diameter of BFs gradually decreased as the opening times increased. Bamboo mainly contained vascular bundles and parenchyma cells, whereas vascular bundles contained fibers, parenchyma cells, and ductal tissues [26]. As the opening times increased, the parenchyma cells on the fiber surface detached, and the fibers gradually separated from the bundles into single fibers (Figure 4). In the vascular bundles of BF-1, numerous single fibers were aggregated, and the fibers were not fully separated after the opening process. The macroscopic morphology of some of the fibers was close to cylindrical, with ductal tissues inside and a substantial number of parenchyma cells on the surface layer. Because BF-2 and BF-3 were opened several times, the surface layer of parenchyma cells was thinner. The size of the bundles shrank as the gathered fibers gradually separated, and the macroscopic morphology of the fibers was similar to a semi-separated cylindrical shape. BF-4 and BF-5 were no longer covered in parenchyma cells, with only a small number of cells remaining. The fiber bundle was basically made of the bamboo’s own fibers gathered together. There was no ductal tissue. The fiber bundle surface was comprised of the parallel arrangement of fibers caused by the straight groove. The size of BF-6 was the smallest and close to the size of bamboo single fibers. There were no parenchyma cells on the fiber surface, and each fiber bundle consisted of several single BFs gathered together in parallel. The macroscopic shape was flat, but the surface showed grooves.

### 3.2. Chemical Compositions Analysis

With the increase in the number of opening processes, the parenchyma cells between BFs were separated and detached by lamination, and more single BFs were retained and tested for their chemical composition. The results are shown in Table 1.

The content of the benzene–ethanol extractive significantly decreased, and the content of other components increased after the rolling and opening treatment, while the contents of the benzene–ethanol extractive and hemicellulose decreased, and the content of cellulose increased with the increase in the number of opening processes of BFs. This was because there were large differences in the chemical composition of bamboo’s vascular bundles and parenchyma cells, and the change in parenchyma-cell content was related to the change in chemical composition. A previous study has shown that the contents of the extractive and hemicellulose in the parenchyma cells of bamboo were significantly higher than those in vascular bundles, while the content of cellulose in parenchyma cells was significantly lower than that in vascular bundles, and the contents of the other components in parenchyma cells and vascular bundles were similar [27]. In this study, BFs were separated by breaking up the fibers as the number of openings increased. The parenchyma cells on the fibers detached, and the proportion of vascular bundles increased, resulting in changes in the chemical composition of BFs. 

The major chemical compositions of bamboo ash are K_2_O (34.23%), SiO_2_ (24.32%), SO_3_ (14.05%), and MgO (6.69%) [28]. Potassium is mainly present in the plant in ionic form and is easily lost due to its high activity. Silica is mainly found in the epidermis, vascular bundles, and vascular sheaths [29]. The destruction of the vascular bundles during mechanical rolling leads to a decrease in silica content. As a result, the ash content of roll-treated BFs is lower than that of untreated bamboo.

**Figure 5:**
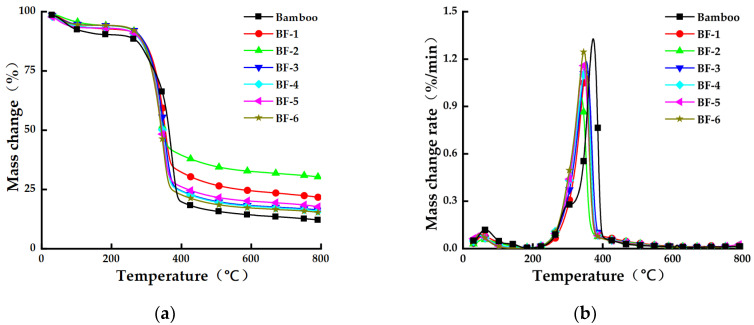
Pyrolysis curves of bamboo fibers with different opening times. (**a**) Mass change–temperature correspondence curve. (**b**) Mass change rate–temperature correspondence curve.

The pyrolysis performance of BFs was tested (Figure 5). The comparative analysis of the TG and DTG curves showed that the pyrolysis of the different BFs all went through three stages, namely, water evaporation, rapid pyrolysis, and slow pyrolysis. The starting pyrolysis temperature band and the maximum rate of the pyrolysis temperature band of different BFs were basically the same at 210 °C–220 °C and 340 °C–360 °C, respectively. With the increase in the opening times, the amount of residual char from BF pyrolysis showed a decreasing trend.

### 3.3. Moisture-Adsorption Characteristics Analysis

#### 3.3.1. Variations in Water Content and Equilibrium Time

**Figure 6:**
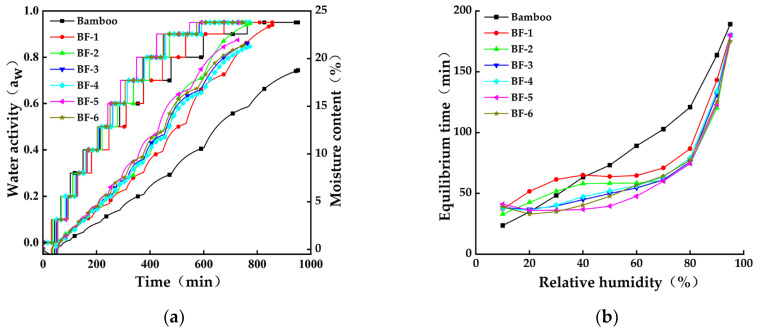
Moisture-adsorption kinetic curve of bamboo fibers with different opening times. (**a**) Water activity and moisture content–time correspondence curve. (**b**) Equilibrium time–relative humidity correspondence curve.

The dynamic moisture-adsorption method was used to obtain the moisture-adsorption kinetic curves of the different fiber sizes at 25 °C (Figure 6). Water is the main factor affecting the moisture-adsorption kinetics of materials. The EMC increased with the increase in the water content, and the moisture content of the specimen increased rapidly, then slowly, until equilibrium at each relative humidity. The smaller the size of the BFs, the longer it took for the specimen to reach 0% moisture. The time required for the bamboo material to reach the EMC was lower than that for the different-sized fibers when the relative moisture content was <40%. This may be related to the structure of the material. The larger the specific surface area of the fiber, the more moisture-adsorption sites, the easier it is to adsorb moisture, and the greater the magnitude of the mass change. However, when the relative moisture content was >40%, it took longer for the bamboo material to reach the EMC. This may be because water clusters are more likely to form in high humidity, especially when the relative humidity is >60%. The structure of BF is looser after mechanical rolling treatment, which is more favorable to the formation of water layers [16]. The untreated bamboo structure, on the other hand, is much tighter; therefore, it takes a long time for water to infiltrate under high-humidity conditions.

As shown in Table 2, the EMCs of the adsorption and the adsorption time at each relative humidity in the curve were analyzed. When they reached equilibrium, the moisture content and the time of the different sizes of BFs were different. At 90% relative humidity, the EMC of all fibers was higher than that of bamboo, which had the lowest EMC at 18.76%. However, it took the longest time to reach equilibrium.

#### 3.3.2. Changes in Hygroscopic-Desorption Process

**Figure 7:**
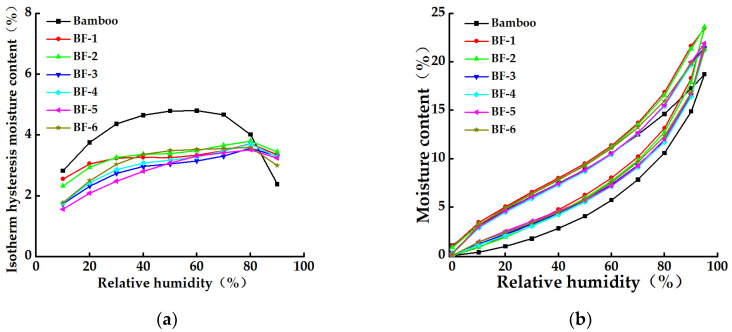
Moisture absorption–desorption curve of bamboo fibers with different opening times. (**a**) Moisture absorption–desorption curve. (**b**) Moisture absorption hysteresis curve.

Further analysis of the moisture absorption–desorption process of rolled and opened BFs is shown in Figure 7.

As shown in Figure 6a, the adsorption curves of bamboo and BFs of different sizes showed a typical S-shaped curve with the characteristics of multi-molecular layer adsorption, and the EMCs of bamboo material and BFs at each stage gradually increased as the relative humidity increased. The EMCs of BF-1 and BF-2 were slightly higher than those of the other BFs. As shown in Figure 6b, hygroscopic hysteresis existed in different materials during the hygroscopic-desorption process, which was mainly related to the rearrangement of molecules in the cell wall of the material and the reduction in the adsorption rate [30]. Hygroscopic hysteresis was the most significant in bamboo, and the difference between the adsorption and desorption EMCs of bamboo at 60% relative humidity was the largest.

This was because the fibers in bamboo were closely arranged, and when BFs were processed by opening, the parenchyma cell cavitied in the closed-pore structure of BFs were exposed, which increased the number of moisture-adsorption sites. Any variation in volume fractions of parenchyma cells can affect the overall hygroscopicity of BFs [31]. BFs with fewer opening times had a higher hemicellulose content and more exposed parenchyma cells cavitied in the fibers. With the increase in the number of openings, the parenchyma cells on the fiber decreased, and the lignin content increased, which reduced the fiber moisture absorption sites and caused a slight decrease in the EMC of the fiber.

#### 3.3.3. Change in EMC Increment

**Figure 8:**
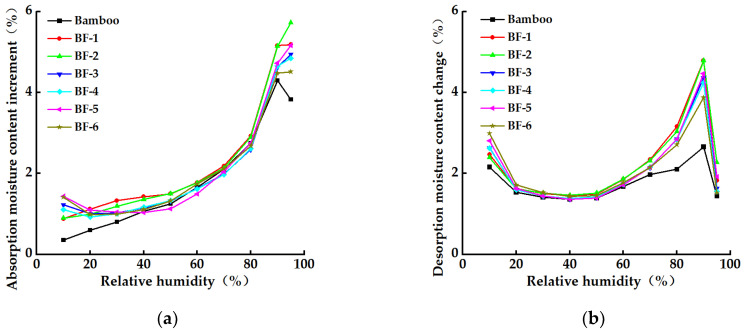
Water content change curve of bamboo fibers during water adsorption. (**a**) Change curve of absorption moisture content. (**b**) Change curve of desorption moisture content.

Figure 8 gives the changes in the EMC increment of bamboo material and BFs during the moisture uptake–desorption process. As shown in Figure 8a, the change in the growth rate of the EMCs of bamboo and BFs during the hygroscopic process was smaller when the relative humidity was between 0% and 50%. This was because, at low relative humidity, the change in the EMCs of the materials mainly occurred on the surface of the hydroxyl water adsorption sites, where the water was arranged in a monolayer. The value of the EMC increment of bamboo timber was lower than that of BF at low relative humidity, indicating that the specific surface area of the bamboo material was smaller. The EMC growth rate of the material increased significantly when the relative humidity was >60% and was the greatest at a relative humidity of 90%. This was because the cell-wall matrix of the BFs swelled more in high relative humidity settings, providing more water adsorption sites. However, because the cell wall has a complicated structure and cannot swell indefinitely, the water content increment decreased at a relative humidity of 95% [24].

As shown in Figure 8b, during desorption, as the relative humidity decreased, the water content of the bamboo material and BFs changed with the trend of the water content changes at both ends. The incremental curve of the EMC showed a U-shaped change, and the loss rate of the EMC decreased when the relative humidity decreased from 90% to 60%. This was mainly due to multilayer moisture adsorption on the surface of the material. The moisture-adsorption force was relatively weak, and when the relative humidity was reduced, more moisture escaped. The change in the EMC loss rate was small during the decrease in the relative humidity from 60% to 20%, and the moisture gradually escaped from the surface of the material. The EMC loss rate was larger in the final stage of desorption when the relative humidity dropped from 20% to 0%. During the whole desorption process, the rate of change of EMC of the bamboo material was the smallest.

#### 3.3.4. Model Fitting and Evaluation of EMC

The EMC of BFs at different relative humidity was fitted by Origin 9.1 software. The GAB-related parameters were obtained, and the evaluation indexes of the analytical fitting effect are shown in Table 3. The coefficient of determination R2 of the GAB model during the absorption of the different materials was >0.99, and the coefficient of determination R2 of the GAB model during desorption was >0.95. The fitting evaluation indexes of the GAB model all achieved the desired effect [32]. The saturated moisture contents of bamboo, BF-1, BF-2, BF-3, BF-4, BF-5, and BF-6 materials were 23.14%, 29.79%, 30.73%, 27.76%, 27.46%, 29.06%, and 27.01%, respectively; BF-1 and BF-2 had the strongest moisture absorption ability.

## 4. Conclusions

In this work, the effects of morphology, chemical composition, and pyrolysis performance were analyzed. The hygroscopic behavior of BFs of different sizes after mechanical rolling and fiber opening was investigated via water-vapor sorption isotherms fitted by the GAB model. The conclusions are as follows:

(1) The morphology and structure of BFs were largely affected by the opening times, with a significant separation of parenchymal cells when more than four treatments were performed. After six treatments, the surface of fibers was essentially free of parenchymal cells.
(2) The size of the BFs affected the moisture-adsorption properties of the material. With the increase in the fiber-opening times, the adsorption strength of water molecules on the fiber material decreased. This led to the observed differences in the hygroscopic behavior and hysteresis of the different samples.
(3) As the relative humidity increased, the cell wall matrix of BF expanded to provide more moisture-adsorption sites, so the EMC gradually became larger. However, the increment in moisture content decreased when the relative humidity exceeded 95% due to the cell wall being unable to expand indefinitely and the limited number of adsorption sites.

This research study provides insights into the hygroscopic mechanism of BFs, and the information generated in this study can provide useful information in optimizing the manufacturing process and modeling the hygroscopic responses of BFs of different sizes.

## Figures and Tables

**Table 1:** Chemical composition of bamboo fibers with different opening times.

Index	Moisture Content (%)	Benzene–Ethanol Extractives (%)	Hemicellulose (%)	Lignin (%)	Cellulose (%)	Ash (%)
BF-1	7.21	3.95	18.80	16.01	57.57	0.52
BF-2	9.76	3.73	17.13	16.62	58.83	0.99
BF-3	7.59	3.51	17.47	16.41	58.89	1.05
BF-4	8.16	2.97	16.81	17.32	59.43	0.98
BF-5	7.40	2.78	15.95	16.65	61.74	0.51
BF-6	6.73	2.51	15.83	17.30	61.81	1.01
Bamboo	8.95	8.99	14.81	13.99	57.47	1.51

**Table 2:** Moisture-adsorption equilibrium parameters of bamboo fibers with different opening times.

Index		Relative Humidity (%)									
		10	20	30	40	50	60	70	80	90	95
Bamboo	Time (min)	66.79	101.66	149.96	213.3	286.48	375.56	478.34	599.16	762.78	951.84
	EMC (%)	0.39	0.99	1.79	2.86	4.12	5.78	7.88	10.64	14.93	18.76
BF-1	Time (min)	68.42	120.01	181.49	246.53	310.32	375.01	445.87	532.61	675.77	855.77
	EMC (%)	0.92	2.04	3.37	4.79	6.29	8.07	10.25	13.18	18.34	23.53
BF-2	Time (min)	68.49	111.1	162.85	220.91	279.19	337.72	398.11	472.41	592.5	772.5
	EMC (%)	0.96	1.96	3.15	4.51	6.01	7.78	9.91	12.81	17.95	23.67
BF-3	Time (min)	88.63	125.64	165.29	210.14	260.1	314.58	375.71	451.76	583.04	763.05
	EMC (%)	1.32	2.33	3.35	4.49	5.81	7.44	9.43	12.02	16.65	21.59
BF-4	Time (min)	83.41	119.53	159.76	206.95	259.05	315.32	378.87	457.94	591.84	771.84
	EMC (%)	1.15	2.08	3.08	4.25	5.59	7.20	9.17	11.78	16.44	21.28
BF-5	Time (min)	93.55	129.63	165.67	202.44	241.93	289.54	349.6	424.13	547.18	727.19
	EMC (%)	1.47	2.57	3.61	4.65	5.78	7.27	9.31	12.07	16.79	21.96
BF-6	Time (min)	94.4	127.31	162.28	202.58	250.28	307.1	371.39	448.76	573.86	748.75
	EMC (%)	1.44	2.43	3.42	4.53	5.85	7.59	9.70	12.38	16.86	21.37

**Table 3:** Parameters of the GAB model for bamboo fibers with different opening times.

Index		W_0_	C	K	R_SS_
Absorption	Bamboo	6.371	0.967	0.785	0.035
	BF-1	5.103	3.001	0.839	0.099
	BF-2	4.662	3.122	0.856	0.128
	BF-3	4.245	4.187	0.853	0.065
	BF-4	4.264	3.645	0.852	0.059
	BF-5	3.964	5.733	0.867	0.042
	BF-6	4.579	3.989	0.838	0.049
Desorption	Bamboo	5.129	−2.3 × 10^45^	0.778	1.694
	BF-1	5.406	−2.5 × 10^44^	0.822	1.777
	BF-2	5.248	3.86 × 10^44^	0.828	1.720
	BF-3	4.961	−2.1 × 10^45^	0.822	1.606
	BF-4	4.928	2.38 × 10^45^	0.821	1.700
	BF-5	4.967	−1.6 × 10^44^	0.825	1.493
	BF-6	5.309	4.51 × 10^44^	0.804	1.748

## Data Availability

All the data have been presented in the manuscript.
